# Impact of cyclic changes in pharmacokinetics and absorbed dose in pediatric neuroblastoma patients receiving [^177^Lu]Lu-DOTATATE

**DOI:** 10.1186/s40658-022-00436-4

**Published:** 2022-03-28

**Authors:** Javian C. Malcolm, Nadia Falzone, Jennifer E. Gains, Matthew D. Aldridge, David Mirando, Boon Q. Lee, Mark N. Gaze, Katherine A. Vallis

**Affiliations:** 1 Oxford Institute for Radiation Oncology, University of Oxford, Old Road Campus Research Building, Roosevelt Drive, Oxford, OX3 7DQ UK; 2 Department of Oncology, University College London Hospitals NHS Foundation Trust, London, UK; 3 Institute of Nuclear Medicine, University College London Hospitals NHS Foundation Trust, London, UK; 4 MIM Software, Beachwood, OH USA

**Keywords:** [^177^Lu]Lu-DOTATATE, Dosimetry, Inter-cyclic changes, Pharmacokinetics, Peptide receptor radionuclide therapy, Neuroblastoma

## Abstract

**Purpose:**

Recent reports personalizing the administered activity (AA) of each cycle of peptide receptor radionuclide therapy based on the predicted absorbed dose (AD) to the kidneys (dose-limiting organ) have been promising. Assuming identical renal pharmacokinetics for each cycle is pragmatic, however it may lead to over- or under-estimation of the optimal AA. Here, we investigate the influence that earlier cycles of [^177^Lu]Lu-DOTATATE had on the biokinetics and AD of subsequent cycles in a recent clinical trial that evaluated the safety and activity of [^177^Lu]Lu-DOTATATE in pediatric neuroblastoma (NBL). We investigated whether predictions based on an assumption of unchanging AD per unit AA (Gy/GBq) prove robust to cyclical changes in biokinetics.

**Methods:**

A simulation study, based on dosimetry data from six children with NBL who received four-cycles of [^177^Lu]Lu-DOTATATE in the LuDO trial (ISRCTN98918118), was performed to explore the effect of variable biokinetics on AD. In the LuDO trial, AA was adapted to the patient’s weight and SPECT/CT-based dosimetry was performed for the kidneys and tumour after each cycle. The largest tumour mass was selected for dosimetric analysis in each case.

**Results:**

The median tumour AD per cycle was found to decrease from 15.6 Gy (range 8.12–26.4) in cycle 1 to 11.4 Gy (range 9.67–28.8), 11.3 Gy (range 2.73–32.9) and 4.3 Gy (range 0.72–20.1) in cycles 2, 3 and 4, respectively. By the fourth cycle, the median of the ratios of the delivered AD (AD_D_) and the predicted (or “expected”) AD (AD_E_) (which was based on an assumption of stable biokinetics from the first cycle onwards) were 0.16 (range 0.02–0.92, *p* = 0.013) for the tumour and 1.08 (range 0.84–1.76, *p* > 0.05) for kidney. None of the patients had an objective response at 1 month follow up.

**Conclusion:**

This study demonstrates variability in Gy/GBq and tumour AD per cycle in children receiving four administrations of [^177^Lu]Lu-DOTATATE treatment for NBL. NBL is deemed a radiation sensitive tumour; therefore, dose-adaptive treatment planning schemes may be appropriate for some patients to compensate for decreasing tumour uptake as treatment progresses.

*Trial registration* ISRCTN ISRCTN98918118. Registered 20 December 2013 (retrospectively registered).

**Supplementary Information:**

The online version contains supplementary material available at 10.1186/s40658-022-00436-4.

## Introduction

Peptide receptor radionuclide therapy (PRRT) which targets the somatostatin receptor SSTR2 using [^177^Lu]Lu-DOTATATE is widely used in the treatment of somatostatin-positive neuroendocrine tumours (NET) in adults with low or moderate Ki67 proliferation index [1]. PRRT either alone or in combination with additional anticancer agents has shown efficacy in a range of other somatostatin-positive cancers [2, 3]. In the prospective NETTER-I Phase III trial, patients with metastatic midgut NET received either 4 cycles of [^177^Lu]Lu-DOTATATE (7.4 GBq) plus octreotide or octreotide alone (control group). Treatment with [^177^Lu]Lu-DOTATATE was associated with improved progression-free survival at 20 months: 65.2% (95% confidence interval [CI] 50.0 to 76.8) versus 10.8% (95% CI 3.5 to 23.0) in the octreotide only group [4].

The use of dosimetry to individually prescribe the number of cycles or administered activity (AA) per cycle each patient receives remains a debated topic for radionuclide therapy, with the approach being increasingly incorporated into national guidelines [5] and recent reports of successful dosimetry-based treatment planning to maximize the tolerable activity within a prescribed renal limit such as 23 Gy [6–9]. Fixed activity schemas remain in use, with AA adapted to the patient’s tumour burden, renal function, weight or other clinical factors before the initial cycle [10, 11]. While pragmatic, this approach does not account for changes in a patient’s tumour load and renal function after non-initial cycles, and the impact that these changes have on the previously planned activity per cycle.

The recently reported “tumour sink effect” highlights an inverse dependence of renal and tumour uptake of [^68^Ga]Ga-DOTATATE in NET patients with SSTR2-positive tumours and of [^177^Lu]Lu-PSMA I&T in prostate cancer patients who underwent a single time point PET-CT for staging [12, 13]. We anticipate that a change in per cycle biokinetics and absorbed dose (AD) may be observed during PRRT if the treatment is successful in reducing tumour load, and an adjusted regimen based on cyclic changes in renal and tumour AD and kinetics may reduce over- or under-estimation of the optimal AA in non-initial cycles and amplify the therapeutic response. However, no study has yet explored whether inter-cycle changes in AD and biokinetics might occur when performing PRRT over four cycles of treatment in SSTR2-positive patients.

Recently, the use of PRRT has been investigated in the treatment of pediatric neuroblastoma (NBL) which often expresses SSTR2 [14, 15]. The [^177^Lu]Lu-DOTATATE (LuDO) trial (ISRCTN98918118) was a Phase IIa, single centre clinical trial to evaluate the safety and activity of [^177^Lu]Lu-DOTATATE in metastatic high-risk relapsed or refractory NBL patients [16]. Please see reference 16 for full details of the trial. The activity per cycle was individually planned for each patient using a fixed activity calculated per patient weight. Using this example, we evaluated whether the ‘sink effect’ was evident over the course of treatment and if the AD predictions of a fixed-activity schema prove robust to cyclic changes in biokinetics and AD.

## Materials and methods

### Patients and therapy

Twenty patients with relapsed or primary refractory NBL were treated with [^177^Lu]Lu-DOTATATE in up to four cycles in the LuDO trial [16]. Eight patients received a complete course (i.e. four cycles) of [^177^Lu]Lu-DOTATATE. For logistic or medical reasons two of these eight patients did not have a complete set of post [^177^Lu]Lu-DOTATATE SPECT/CT scans. However, full imaging and dosimetric data were available for the remaining six patients (mean age 7.7 years, range 4–11 years), who form the basis of the current study. At baseline all patients had bone metastases and an adrenal primary site. Patient characteristics are shown in Table 1.

**Table 1:** Summary of patient characteristics and personalized [^177^Lu]Lu-DOTATATE therapy (*n* = 6)

Patient	Age at first treatment cycle (years)	Sex	GFR (mL min^−1^)	Baseline weight prior to each cycle (kg)	Per cycle administered amount of radioactivity (GBq)	Per cycle administered amount of radioactivity per weight (MBq/kg)
P1	10	F	144	37.2, 42.8, 42.8, 44.0	2.86, 3.67, 4.68, 4.25	76.9, 85.7, 109.3, 96.6
P2	6	M	77	19.0, 22.6, 23.6, 25.4	1.45, 2.16, 2.57, 2.86	76.3, 95.6, 109, 113
P3	5	M	115	21.4, 24.2, 25.8, 27.1	1.54, 2.00, 2.07, 2.15	71.9, 82.6, 80.2, 79.3
P4	10	F	122	33.0, 35.7, 37.2, 38.2	2.55, 3.61, 3.54, 3.60	77.2, 101, 95.2, 94.2
P5	4	M	83	20.0, 19.6, 21.0, 21.8	1.55, 2.08, 2.04, 2.39	77.5, 106, 97.1, 110
P6	11	F	122	34.5, 39.5, 42.9, 46.1	3.05, 4.11, 3.70, 4.71	88.4, 104, 86.2, 102

### Administration and imaging of [^177^Lu]Lu-DOTATATE

The prescription of [^177^Lu]Lu-DOTATATE was based on an intended weight-adjusted clinical protocol of 75 MBq/kg for the first cycle which was escalated to 100 MBq/kg for the remaining cycles provided there was no renal or hematological toxicity and that the whole-body AD was less than 0.5 Gy. Previous weight measurements were used as the radiopharmaceutical had to be ordered at least two weeks in advance of delivery. Patient weight varies over time, and sometimes the weight at the time of administration was significantly different from that at the time of ordering the radiopharmaceutical. This means that the actual measured AA in relation to the weight at the time of treatment was sometimes different from the intended AA of 75 or 100 MBq/kg, but the aim was to be within ± 10%. Cycles were given at intervals of 8 to 10 weeks. To reduce the radiation AD to the kidneys, an intravenous (i.v.) infusion of amino acids (2.5% l-lysine HCl and 2.5% l-arginine in water for injection) at the rate of 1 L over 4 h was commenced 30 min before the administration of [^177^Lu]Lu-DOTATATE. An infusion of [^177^Lu]Lu-DOTATATE was then administered i.v. over 30 min. All children were hydrated for 24 h along with administration of [^177^Lu]Lu-DOTATATE.

SPECT/CT images of the upper abdomen including organs at risk (kidneys, liver and spleen) were acquired at 4, 24, 48 and 72 h post injection (p.i.) to map the pharmacokinetic uptake and clearance. Imaging was performed using a GE Discovery NM/CT 670 (GE Healthcare, Waukesha, USA) dual-headed gamma camera equipped with 3/8" thick NaI(Tl)-crystals. Camera calibration was performed using a water filled phantom of known volume with known activity (calibrator traced to National Physical Laboratory standard). The phantom was imaged under clinical conditions to derive a planar and SPECT sensitivity factor (cps/MBq). Partial volume correction was performed using an image quality phantom with spheres of varying volumes to generate a recovery curve, and therefore apply a partial volume correction to smaller lesions evaluated during the dosimetric analysis. Phantom experiments were performed under clinical conditions identical to conditions for patient data acquisition and included scatter and attenuation correction. The image acquisition parameters are summarized in the Additional file  1: Table S1. Triple energy window scatter correction was performed over the 208 keV emission window with scatter windows at 170 keV (± 8%) and 244 keV (± 6%). The acquired SPECT data were iteratively reconstructed using Ordered Subsets Expectation Maximization (OSEM) (two iterations, ten subsets) with CT-based correction for attenuation using low-dose CT (20 mA, 120 kV, 1.25 mm slice thickness).

### Cyclic changes in absorbed dose and kinetics

After each cycle the largest tumour mass, which was the primary adrenal tumour in 5 out of 6 cases and a large sacral bone metastasis in one case, was selected for segmentation using the low dose CT scan. Tumour and kidney segmentation was performed at each timepoint and was confirmed by a senior radiologist. The delineated tumours were visible on the CT at each time point in a cycle. MIM SurePlan MRT (MIM Software Inc.) was used to deformably register and convert each SPECT image into an absorbed dose rate map using the kernel convolution method. The alignment of the segmentation between different time points was checked through visual inspection and adjusted as needed. Absorbed dose rate images were calculated for each time point rather than the integrated AD from a cumulated activity map. The effective half-life (*T*_eff_) was obtained by fitting a mono-exponential function to the aligned 24, 48 and 72 h absorbed dose rate images. The AD map was calculated by adding the area under the absorbed dose rate curve corresponding to the time of injection, with 4 h and 24 h time points found by trapezoidal numerical integration followed by integration of the mono-exponential from 24 h to infinity. The integration from 24 h to infinity is shown in Eq. 1:

1 $$D = \dot{D}_{{{24}\,{\text{h}}}} \times \frac{{T_{{{\text{eff}}}} }}{{{\text{ln}}\left( 2 \right)}} {\text{for}} \,t \ge 24\,{\text{h}}$$

where $T_{{{\text{eff}}}}$ is the effective half-life and $\dot{D}_{{{24}\,{\text{h}}}}$ is the absorbed dose rate at the 24 h scan. The renal AD and half-life were calculated as the mean of the left and right kidneys in all patients for each cycle. The relative difference (in %) between AD per unit AA (Gy/GBq), AD (Gy) or effective half-life (h) of the first and non-initial cycles was defined as follows:

2 $$\Delta \left( \% \right) = 100 \times \frac{{V_{{{\text{NI}}}} - V_{1} }}{{V_{1} }}$$

where $V_{{{\text{NI}}}}$ is the AD per unit AA, AD or effective half-life for a non-initial cycle and $V_{1}$ is the value for the initial cycle.

### Simulation of a fixed absorbed dose per unit of administered activity schema

The predictability of a dosimetry protocol for non-initial cycles assuming the same biokinetics as the initial cycle was investigated. This was intended to simulate the common clinical dosimetry practice of assuming invariable biokinetics to predict per-cycle dose as an alternative to full dosimetry after each cycle. Stable biokinetics were simulated by using the AD per unit AA (Gy/GBq) value that was obtained for the first cycle and applying it to subsequent cycles. The expected AD for cycles 2, 3 and 4 (${\text{AD}}_{E}^{{{\text{C}}2,3,4}} )$ was calculated using the following equation:

3 $$\left( {{\text{AD}}} \right)_{{\text{E}}}^{{{\text{C}}2,3,4}} \left( {{\text{Gy}}} \right) = \left( {\frac{{{\text{AD}}}}{{{\text{AA}}}}} \right)_{D}^{{{\text{C1}}}} ({\text{Gy/GBq}}) \times \left( {{\text{AA}}} \right)_{{\text{D}}}^{{{\text{C}}2,3,4}} \left( {{\text{GBq}}} \right)$$

where $\left( {\frac{{{\text{AD}}}}{{{\text{AA}}}}} \right)_{{\text{D}}}^{{{\text{C1}}}}$ is the AD/AA delivered in the first cycle and $\left( {{\text{AA}}} \right)_{{\text{D}}}^{{\text{C2,3,4}}}$ is the AA delivered in cycles 2,3 and 4. The median of the individual ratios of delivered to expected AD for each patient was calculated for each non-initial cycle.

### Response analysis

Overall response was evaluated in the trial using the International Neuroblastoma Response Criteria (INRC) [17, 18] based on investigations one month after the fourth cycle of [^177^Lu]Lu-DOTATATE. However, overall response is not a good assessment of the response of an individual lesion, and so in addition, for this paper, we have also reported changes in the primary lesion using RECIST criteria [19].

### Statistical analysis

All continuous data are expressed as the median and range. The difference in AD and effective half-life between cycles was tested for significance using a two-tailed Wilcoxon-Mann–Whitney test. Spearman *r* and *p* values were computed to assess the strength of the correlation between the estimated AD and effective half-life for each cycle for the tumour and kidneys. All statistical analyses were performed using Excel and *p* values of < 0.05 were considered statistically significant.

## Results

### Cyclic changes in absorbed dose and kinetics

**Fig. 1:**
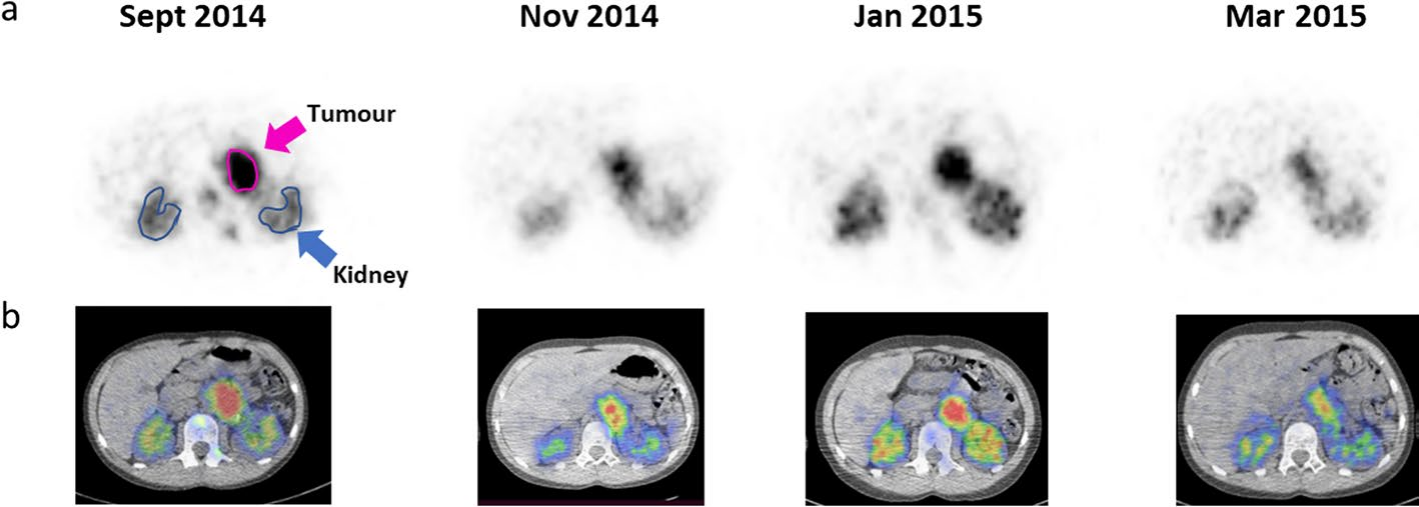
Longitudinal SPECT images at 24 h after each cycle of [^177^Lu]Lu-DOTATATE showing change in uptake of lesion over 24 weeks for Patient 1 as seen on the axial **a** SPECT and **b** SPECT/CT slices

**Fig. 2:**
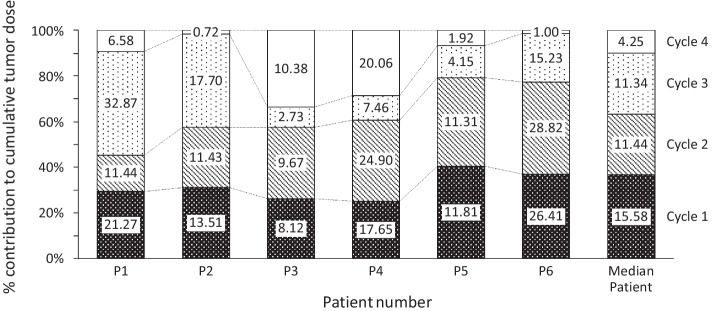
The contribution to the cumulative absorbed dose to the tumour from each cycle of therapy for each of the six patients (P1-P6), with the median values shown in the last bar. The numbers in the bars represent the absolute values of the tumour absorbed dose in Gy

SPECT images (24 h after each of 4 cycles) are shown for a representative patient (patient P1) in Fig. 1. The median tumour AD per cycle was found to decrease as treatment continued with values of 15.6 Gy (range 8.12–26.4), 11.4 Gy (range 9.67–28.8), 11.3 Gy (range 2.73–32.9) and 4.3 Gy (range 0.72–20.1) for cycles 1, 2, 3 and 4, respectively. The individual per cyclic dosimetry results are summarized in Table 2 where the deviation in tumour AD across cycles within the same patient is evident. Variation in AD per unit AA (Gy/GBq) is shown in Additional file 1: Table S2. The greatest difference from the initial cycle for tumour AD was seen in patient P2 with a greater than 90% difference between cycles 1 and 4. The first 2 treatment cycles contributed more than 50% (range 57.5—79.2%) to the cumulative tumour AD in 5 out of 6 patients (Fig. 2**).** As an illustrative example we can compare patients P1, P4 and P6 who received total AD within ± 2 Gy with very different median contributions from the first two cycles (45.3%, 60.7%, 77.3%)**.**

**Table 2:** Per cycle dosimetry, effective half-life and response one month after final cycle for all patients

Patient^‡^	Per cycle				Cumulative		Follow up
	Tumour absorbed dose (Gy)	Tumour effective half-life (h)	Kidney absorbed dose (Gy)	Kidney effective half-life (h)	Tumour absorbed dose (Gy)	Kidney absorbed dose (Gy)	Δ in tumour dimension
P1-1	21.3	54.0	2.60	85.5	21.3	2.6	–
P1-2	11.4	39.0	3.18	71.3	32.7	5.78	–
P1-3	32.9	26.5	1.47	43.8	65.6	7.25	–
P1-4	6.6	31.2	4.02	47.0	72.2	11.27	+ 4.65%
P2-1	13.5	43.6	1.00	109.5	13.5	1	–
P2-2	11.4	37.8	1.03	102.0	24.9	2.03	− 1.86% *
P2-3	17.7	20.0	1.66	69.8	42.6	3.69	–
P2-4	0.72	29.8	1.81	73.4	43.32	5.5	–
P3-1	8.12	51.0	1.28	153.1	8.12	1.28	–
P3-2	9.67	37.7	1.19	93.4	17.79	2.47	–
P3-3	2.73	24.8	1.28	87.9	20.52	3.75	–
P3-4	10.4	25.9	1.98	118.4	30.92	5.73	− 5.47%
P4-1	17.7	40.9	1.53	39.8	17.7	1.53	–
P4-2	24.9	40.3	1.59	82.4	42.6	3.12	–
P4-3	7.5	22.9	2.87	86.7	50.1	5.99	–
P4-4	20.1	35.5	2.82	57.8	70.2	8.81	+ 4.26%
P5-1	11.8	57.8	0.66	91.7	11.8	0.66	^a^
P5-2	11.3	47.6	0.66	132.9	23.1	1.32	^a^
P5-3	4.15	25.8	1.77	95.0	27.25	3.09	^a^
P5-4	1.92	35.1	1.79	26.9	29.17	4.88	^a^
P6-1	26.4	56.2	2.32	102.5	26.4	2.32	–
P6-2	28.8	43.8	3.20	146.1	55.2	5.52	–
P6-3	15.2	30.5	3.03	52.0	70.4	8.55	–
P6-4	1.00	32.5	3.03	100.6	71.4	11.58	− 2.08%
Median	11.4	36.6	1.78	87.3	30.0	3.72	

^a^Patient 5 had no measurable primary lesion at baseline

*Data not available following cycle 4

^‡^Format is [patient number-cycle number]

**Fig. 3:**
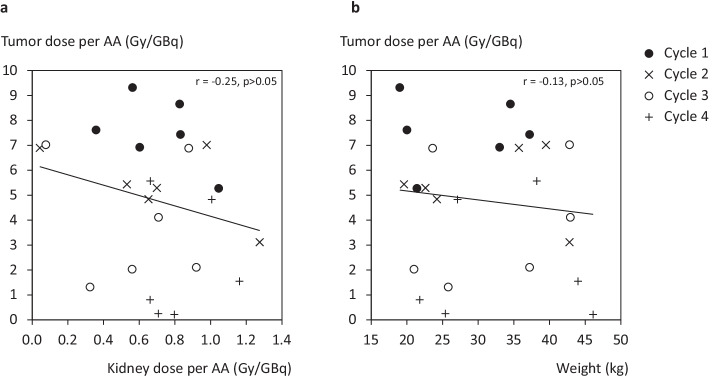
Plot of tumour absorbed dose per unit AA (Gy/GBq) vs **a** the kidney absorbed dose per AA and **b** patient weight are shown as a function of individual treatment cycle. No correlation observed

Normalizing for the AA, the differences between tumour AD per unit AA in cycles 1 and 2 were not statistically significant (median − 23.8%; range − 58.1 to 0%); however, there was a significant difference between cycles 1 and 4 (median 84.3%, range − 8.4 to 97.5%)**.** The ratio of effective half-lives was analysed and it was found that there was no significant difference (*r* = − 0.36, *p* = 0.08) between the effective half-life estimated in the first cycle relative to the final cycle for the kidneys with the values being either constant or showing a slight decrease. There was no trend toward an increase or decrease seen in the intermediate cycles (2 and 3) of the kidneys and tumour within the same patient. There was a moderate negative correlation (*r* = − 0.25, *p* = 0.232) between renal and tumour AD per unit AA using data from all cycles. Visual inspection of Fig. 3a indicates a changing relationship as treatment continues; however, the sample size per cycle (*n* = 6) was too small for inter-cycle correlation analysis. There was no significant correlation (*r* = − 0.13, *p* > 0.05) between the tumour AD per unit AA and patient weight as shown in Fig. 3b.

### Simulation of a fixed absorbed dose per unit administered activity schema

**Fig. 4:**
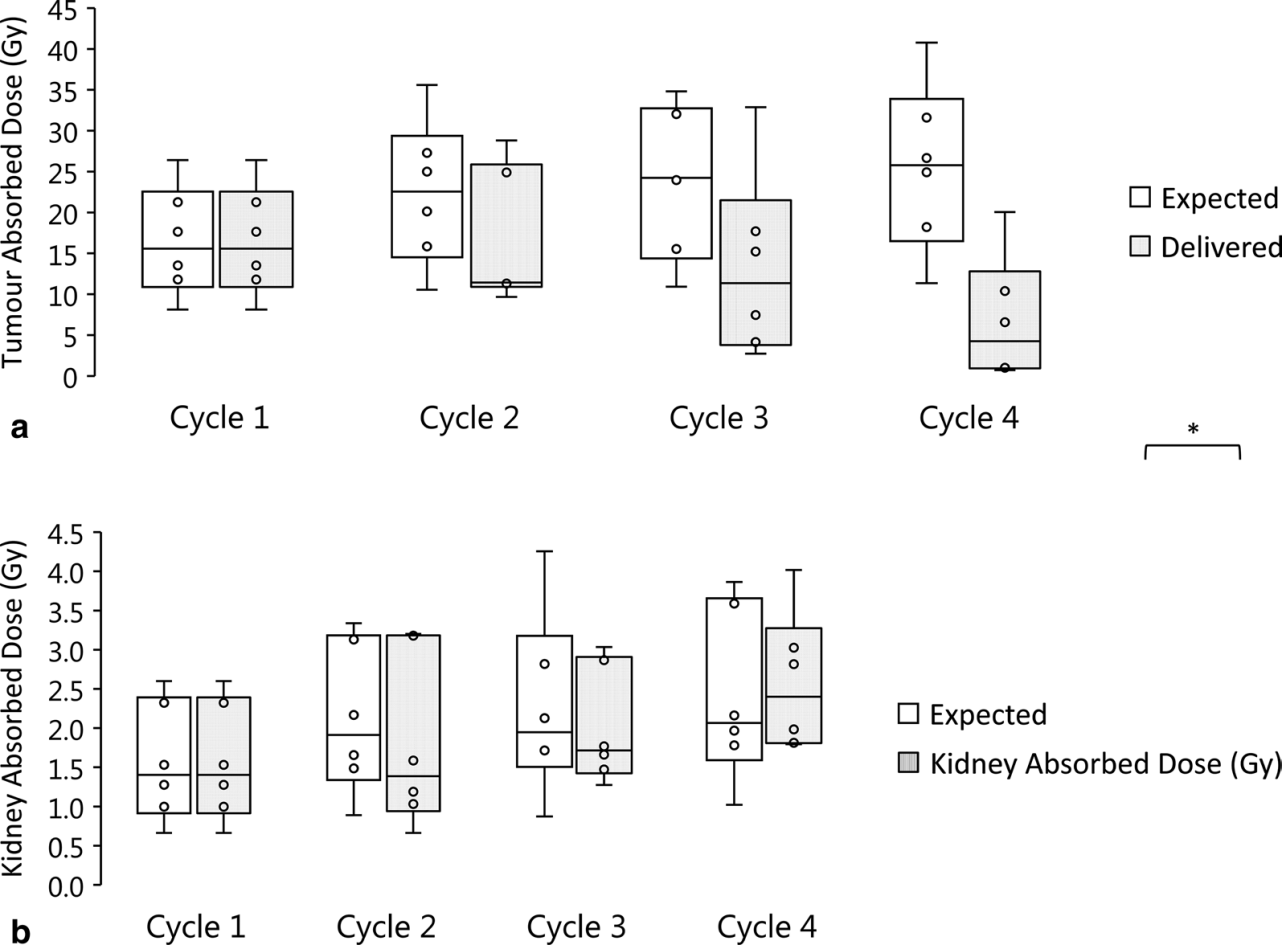
Per-cycle comparison between the expected absorbed dose (AD_E_) in Gy (when based on a fixed-AD per AA scheme) and the delivered absorbed dose (AD_D_) for all six-patients (P1-P6) for **a** the tumour and **b** the kidneys

The median of the individual patient ratios (AD_D_/AD_E_) (where AD_E_ assumes AD per unit AA did not change after the first cycle) is summarized in Table 3 and Fig. 4. It is of note that we found no statistical significance (*p* > 0.05) between the renal expected and delivered ADs in all cycles indicating patients received similar AD to the expected one. However, the fixed AD per unit AA schema progressively over-estimated the AD delivered to the tumour with a statistically significant difference by cycle 4 (*p* = 0.013). Accordingly, the median ratio (AD_D_/AD_E_) for the tumour was 0.76 (0.42–1.00) in the second cycle, 0.39 (0.25–0.94) by the third and 0.16 (0.02–0.92) by the fourth. Patients P2, P5 and P6 showed a striking underestimation of the cumulative tumour AD by more than 50% as predicted from the initial cycle of therapy.

**Table 3:** Summary of expected absorbed dose (based on the assumption of invariable uptake after the first cycle) compared to the delivered absorbed dose (*n* = 6) for the tumour and kidney

	Absorbed dose per cycle (Gy)			
	Expected assuming fixed biokinetics, AD_E_	Delivered, AD_D_	Ratio (AD_D_/AD_E_)	*p* value
Tumour				
Cycle 1	15.6 (8.10–26.4)	15.6 (8.1–26.4)	1	–
Cycle 2	22.6 (10.6–35.6)	11.4 (9.7–28.8)	0.76 (0.42–1.00)	*p* > 0.05
Cycle 3	24.2 (10.9–34.8)	11.3 (2.7–32.9)	0.39 (0.25–0.94)	*p* > 0.05
Cycle 4	25.8 (11.3–40.8)	4.3 (0.7–20.1)	0.16 (0.02–0.92)	0.013
Kidney				
Cycle 1	1.40 (0.66–2.60)	1.40 (0.66–2.60)	1	–
Cycle 2	1.91 (0.89–3.34)	1.39 (0.66–3.20)	0.74 (0.69–1.02)	*p* > 0.05
Cycle 3	1.95 (0.87–4.26)	1.71 (1.28–3.03)	1.01 (0.35–2.03)	*p* > 0.05
Cycle 4	2.06 (1.02–3.86)	2.40 (1.79–4.02)	1.08 (0.84–1.76)	*p* > 0.05

Data are presented as median (range)

### Absorbed dose–response relationship

All five patients with RECIST-evaluable disease had stable disease. In Patient 5 the lesion used in this study was a bone metastasis and therefore not evaluable for response. The change in tumour dimension at one month follow-up is shown in Table 2. No objective response was seen in any of the patients.

## Discussion

To optimize planning of multi-cycle [^177^Lu]Lu-DOTATATE treatments, it is important that the prescribed activity based on dosimetric measurements before or after the first cycle is valid for subsequent cycles. As shown in the current results, it is a valid approximation to assume stable (or sufficiently similar) kidney AD per unit AA between the first and final cycle when planning personalized [^177^Lu]Lu-DOTATATE, as first proposed by Garske et al. [20]. This study expands on this by highlighting that the tumour AD per unit AA decreases with successive cycles. This leads to an uneven contribution to cumulative tumour AD unlike the expected 25% contribution for each cycle that would be predicted from a fixed-activity kidney-based AD prescription. In fact, in this study, the first two cycles contributed more than 50% of the AD (up to 79.2%, median, 63.2%) in all but one patient (P1). The results showed no trend towards higher or lower values in the intermediate cycles (i.e. cycles 2 and 3) in comparison to cycle 1, but rather a clear uneven contribution. The kidney cumulative ADs were well below 23 Gy suggesting an opportunity to increase the AA between cycles without exceeding the threshold kidney dose of 23 Gy. However, there is a need to establish safe limits of renal AD in paediatric patients, especially when there may be a degree of impaired renal function resulting from earlier treatment. We report a negative correlation (*r* = − 0.25, *p* = 0.232) between renal and tumour AD per AA using data from all cycles, implying a trade-off between the tumour and kidney AD. However, this analysis did not reach statistical significance and should be repeated in a larger sample to increase the statistical power in detecting small differences. Interestingly, we did not directly observe variability in kidney uptake as a function of tumour burden which has been observed, for example, in prostate cancer patients treated with [^177^Lu]Lu-PSMA-167 or imaged with [^68^Ga]Ga-PSMA-11 [21, 22]. This could be directly due to the lack of objective responses in this study implying no significant change in tumour burden.

While scanning beyond 72 h could potentially improve the accuracy of this dosimetry approach, the 72 h time-point constitutes a good trade-off between practicality when imaging young children and achieving a clinically relevant level of accuracy. Although the imaging protocol did not include a later time point, the renal effective half-life (median 87.3 h) in this study was only slightly higher than the previously reported values for adult NET [23, 24].

There are various methods available to fit AD rate maps to determine the dose-rate curve and ultimately the absorbed dose. The method that is commonly used in the clinic is a mono-exponential fit, chosen for its simplicity. However, noise in the SPECT/CT acquisition process introduces additional challenges and in general, if the number of imaging time points ≤ 5, a numerical integration may prove more accurate than exponential fits. We elected to use a hybrid integration protocol of numerical trapezoidal integration to 24 h followed by mono-exponential decay beyond 24 h, as it gives similar results to more elaborate biexponential fitting (differences smaller than 5%) while still being practical for routine clinical practice [25].

While feasible in a single-study sponsored trial, repeated SPECT/CT acquisitions after each cycle as described in this study may be considered impractical and too resource-intensive in many healthcare facilities. The recent rise in simplified dosimetry approaches, suggested for normal organs such as kidneys, using a limited number of image acquisitions, may give reasonable accuracy and may allow adaption of the PRRT prescription to changing tumour uptake and clearance at intermediate cycles (i.e. biologically adaptive radionuclide therapy) in routine clinical practice. However, the response and behaviour of individual tumour lesions may be associated with more variation [26]. Single-time point dosimetry is possible but results must be interpreted with caution due to variation in both intra- and inter-patient clearance patterns [9, 27]. The optimal approach may be full dosimetry analysis after Cycle 1 to determine the personalised clearance pattern, followed by single-time point imaging (at 24 h) for subsequent cycles based on the data from Cycle 1.

The causes of inter-cycle variability were not investigated in this study and remain unclear, with no conclusive explanation offered in the literature. Among other factors, changes in tumour radiosensitivity, interstitial pressure and necrosis are likely to influence binding affinity and SSTR2 receptor density and ultimately the capacity of cancer cells to accumulate radioactivity in later cycles. We also acknowledge partial volume effects, respiratory motion and differences in patient positioning during scans may contribute to inter-cycle AD variability. While cyclic changes could result from measurement error, the method used in this study yields reproducible results with acceptable accuracy (median error < 10%) [27].

Typically, prescription of [^177^Lu]Lu-DOTATATE focuses on achieving the maximum tolerable AD which means concentrating on an acceptable safe AD to the kidneys (and to a lesser extent the bone marrow). In this study, a weight-adjusted prescription was used with a whole-body AD limit of 0.5 Gy for this vulnerable paediatric group. In adults, it may seem intuitive to base prescriptions on the AD to the tumour as is done in EBRT. However, unlike EBRT, tumour AD from AA is based on a complex interplay of patient-specific pharmacokinetics. The results from this study suggest that understanding the pharmacokinetics after the first cycle does not necessarily lead to understanding of the pharmacokinetics after subsequent administrations.

NBL, and NETs in general, are relatively radiation sensitive tumours [28]. However, decreasing tumour uptake in later cycles suggests that cyclic changes could lower [^177^Lu]Lu-DOTATATE concentration in the tumour and ultimately likelihood of response. It may be appropriate to deliver the typical four-cycle prescription regimen in either fewer cycles or with higher AA in the earlier cycles. While not every patient is likely to benefit, patients with poor renal function (as measured by eGFR) or high disease burden (as measured by PET-CT) may benefit from additional monitoring over the course of their multi-cycle [^177^Lu]Lu-DOTATATE treatment. The results of the current study support a current NOPHO (Nordric Society of Paediatric Haematology and Oncology) trial, LuDO-N (NCT04903899), which is designed to investigate AD intensification.

In the LuDO trial, [^177^Lu]Lu-DOTATATE monotherapy was not found to be effective in NBL patients by INRC criteria at the AD schedule used. The patients in this dosimetric sub-study however had stable disease by RECIST measurement of the primary tumour. It is important to note that INRC gives an overall response at a defined time point, based on a combination of anatomical and functional imaging, bone marrow assessment and (in earlier versions) tumour markers. It is not an assessment of lesional response. So while INRC is helpful for guiding the overall management of patients, it is not necessarily suitable for the assessment of response to molecular radiotherapy. The lack of objective response may be a result of treatment-resistant tumours or the clinical heterogeneity of this group of patients who were heavily pre-treated. It is, therefore, a shortcoming of the present study that the relationship between cyclic changes in pharmacokinetics and objective response could not be investigated.

There are several possible approaches to improve the efficacy of [^177^Lu]Lu-DOTATATE in NBL patients in future trials. One is to explore more AD-intense schedules, giving higher AA in fewer fractions over a shorter overall time, with personalization of the schedule to deliver a total activity approaching, but not exceeding, the tolerance of critical organs at risk like the kidney. This approach is supported in the LuDO-N trial (NCT04903899)). Another would be to explore combinations with [^131^I]I-mIBG as the differing mechanisms of the two radiopharmaceuticals may potentially overcome limitations of single agent treatment [15, 29].

We acknowledge that the sample size of this exploratory study is limited, and the results must be interpreted with caution. Serial SPECT scans over 3–4 days—termed “full dosimetry”—after each cycle of treatment is generally considered resource intensive in terms of clinical staff hours and scanner time. Therefore, studies like this, relying on full dosimetry after each cycle to track changes in AD and kinetics, are limited by patient numbers. This is compounded by NBL being a rare cancer accounting for only about 6% of all cancers in children. Nevertheless, the results point towards the tumour being under-dosed compared to the predictions from a fixed AD per unit AA dosimetry regimen which assumes stable biokinetics throughout the course of treatment. As new agents such as [^177^Lu]Lu-OPS201 ([^177^Lu]Lu-DOTA-JR11) are developed and introduced, a systematic characterization of changing tumour and kidney AD during successive treatment cycles could add valuable insight for patient prescription individualization.

## Conclusion

In this study, a decreasing contribution to tumour AD with successive treatment cycles was demonstrated coupled with a relatively constant kidney effective half-life. Patient-specific dosimetry simulations based on data from a multi-cycle [^177^Lu]Lu-DOTATATE clinical trial demonstrate the importance of changes in inter-cycle tumour AD and biokinetics in personalizing patient administered activity especially in children.

## Supplementary Information

**Additional file 1.** The Supplementary Information contains a summary of the key specifications of SPECT-CT image acquisition and reconstruction used in this study, and a summary of the per-cycle renal and tumor dose rates (Gy/GBq).

## Data Availability

Not applicable.
