# Supplementary material for: Impact of cyclic changes in pharmacokinetics and absorbed dose in pediatric neuroblastoma patients receiving [177Lu]Lu-DOTATATE
Source: EJNMMI Phys. 2022 Mar 28;9:24. doi: 10.1186/s40658-022-00436-4 (PMC8960523; doi:10.1186/s40658-022-00436-4)
Supplement: Supplementary file 1 — Additional file 1. The Supplementary Information contains a summary of the key specifications of SPECT-CT image acquisition and reconstruction used in this study, and a summary of the per-cycle renal and tumor dose rates (Gy/GBq). [file 40658_2022_436_MOESM1_ESM.docx]

**Supplementary Information**

Impact of Cyclic Changes in Pharmacokinetics and Absorbed Dose in Pediatric Neuroblastoma Patients Receiving [^177^Lu]Lu-DOTATATE

Malcolm JC *et al*.

**Table S1**: Summary of the key specifications of SPECT-CT image acquisition and reconstruction

| **Parameters** | **Value** |
| --- | --- |
| **Acquisition** |  |
| Collimator  Matrix size (pixels) | Medium-Energy General Purpose  128 x 128 |
| Pixel size (mm) | 4.46 x 4.46 |
| Acquisition time | 60 projections x 30s |
| Energy window centered at 208 keV | 20.00 % |
| System sensitivity in 208 keV window (cps/MBq) | 13 |
| Crystal thickness (inches) | 8/8 |
| Slice Thickness (mm) | 4.46 |
| kVp | 120 |
| Tube current (mA) | 100 |

| Focal Spot Size (cm) | 0.70 |
| --- | --- |
| **Reconstruction** |  |

|  |  |
| --- | --- |

| Iterative Method | OSEM* |
| --- | --- |
| Number of iterations | 2 |
| Number of subsets | 10 |

OSEM: Ordered Subsets Expectation Maximization

**Table S2:** Summary of the per-cycle renal and tumor dose rates (Gy/GBq)

| **Patient** | **Organ** | **Cycle1 (Gy/GBq)** | **Cycle 2 (Gy/GBq)** | **Cycle 3 (Gy/GBq)** | **Cycle 4 (Gy/GBq)** |
| --- | --- | --- | --- | --- | --- |
| P1 | tumour | 7.44 | 3.12 | 7.02 | 1.55 |
| P2 | tumour | 9.32 | 5.29 | 6.89 | 0.25 |
| P3 | tumour | 5.28 | 4.84 | 1.32 | 4.83 |
| P4 | tumour | 6.92 | 6.90 | 2.11 | 5.57 |
| P5 | tumour | 7.62 | 5.44 | 2.03 | 0.81 |
| P6 | tumour | 8.66 | 7.01 | 4.12 | 0.21 |
| P1 | kidney | 0.91 | 0.87 | 0.31 | 0.94 |
| P2 | kidney | 0.69 | 0.48 | 0.65 | 0.63 |
| P3 | kidney | 0.83 | 0.59 | 0.62 | 0.92 |
| P4 | kidney | 0.60 | 0.44 | 0.81 | 0.78 |
| P5 | kidney | 0.43 | 0.32 | 0.87 | 0.75 |
| P6 | kidney | 0.76 | 0.78 | 0.82 | 0.64 |
